# Takayasu Arteritis Presenting As Epileptic Seizure: A Case Report

**DOI:** 10.7759/cureus.26520

**Published:** 2022-07-02

**Authors:** Ramesh Shrestha, Abnish Pandit, Ghanshyam Kharel

**Affiliations:** 1 Internal Medicine, Tribhuvan University/Chitwan Medical College, Kathmandu, NPL; 2 Neurology, Chitwan Medical College and Teaching Hospital, Chitwan, NPL; 3 Neurology, Tribhuvan University Institute of Medicine, Kathmandu, NPL

**Keywords:** transient ischemic attacks, takayasu arteritis, vascular disease, ocular ischemic syndrome, cns manifestations, vasculitis, granulomatous vasculitis, pulseless disease

## Abstract

Takayasu's arteritis is a chronic granulomatous large-vessel vasculitis condition that affects the large and medium-sized arteries, primarily the heart and its major vessels. The first symptoms and indicators of Takayasu arteritis differ because the afflicted arteries are heterogeneous. Furthermore, vascular lesions might be difficult to identify at first, further complicating diagnosis. Takayasu arteritis presenting as epileptic seizures is rare. Here, we discuss a 20-year-old female who presented with a brief period of unresponsiveness, followed by a tonic stiffening, limb jerks, a postictal period of fatigue, and temporal memory loss. During the acute phase of Takayasu arteritis, high-dose glucocorticoid therapy and immunosuppressive therapy were used to control inflammatory reactions. Her symptoms gradually improved, and she was discharged from the hospital after serial monitoring; her follow-up visits revealed no recurrence.

## Introduction

Takayasu's arteritis (TA) is a chronic granulomatous large-vessel vasculitis condition affecting the large and medium-sized arteries, most usually affecting the heart and its major vessels [1]. While the extracranial neurovascular signs of TA are well known, the intracranial manifestations are less understood [2]. At first, vascular lesions might be difficult to detect, making diagnosis more challenging. TA is characterized by fatigue, malaise, fever, night sweats, joint aches, weight loss, and fainting at first followed by a pulseless phase. It can also appear as a brief period of unresponsiveness followed by a fall, tonic stiffening, and limb jerks. A postictal period with fatigue, incontinence, and temporal memory loss is possible. However, seizures are rarely the initial clinical symptom [3]. It involves the aorta and its principal branches, but coronary arteries have been shown to be involved in 7% to 9% of cases [4]. Similarly, 7.8% of patients had cerebral vasculitis, and 3.9% with strokes caused by large-vessel obstruction [2]. The most effective treatment for TA is glucocorticoids. Two-thirds of patients, however, have illness recurrence or steroid reliance. As a result, additional immune suppressants are frequently utilized as a supplement or alternative to corticosteroids [5].

## Case presentation

A 20-year-old woman was presented to our center with complaints of generalized abnormal body movement, tonic stiffening, limb jerks, and losing consciousness for 5 to 15 minutes. She was also experiencing headaches and postictal disorientation. For four months, she had headaches and left-sided vision loss. Her headaches lasted an average of one to two hours without an aura or vomiting. She had been experiencing infrequent limb weakness and discomfort for the previous 1.5 years. She did not have a fever, rash, trauma, cough, joint pain, or weight loss. There were no other serious comorbidities or long-term pharmaceutical use. She did not consume alcohol or smoke cigarettes.

On systemic examination, the right carotid artery had carotid bruits and no right radial pulses. Furthermore, the blood pressure in the right upper limb was unrecordable, but it was 100/60 mm Hg in the left arm. The heart rate was 120 beats per minute, the trachea was in the midline position, and the apical impulse was in the left 5th intercostal space. Aortic auscultation revealed a non-radiating systolic murmur. A comprehensive neurological examination revealed no focal neurological deficits, such as stiffness or dyskinesia. Ocular examination indicated 6/60 visual acuity in the right eye (RE) and 6/6 in the left eye (LE). Ophthalmoscopy revealed an obstructed lower temporal branch of a branch retinal artery in RE. During a fundoscopy, the peripapillary retinal nerve fiber layer is edematous, positive hard exudates, and a flame-shaped hinge over the right retina was discovered. The erythrocyte sedimentation rate (ESR) was 59 mm in the first hour of routine hematological examination using the Westergren method, and the C-reactive protein was 44.25 mg/L. The blood sugar level, serum sodium, potassium, urea, and creatinine, as well as other biochemical tests, were within normal limits. The Venereal Disease Research Laboratory (VDRL), anti-streptolysin O (ASO), and antinuclear antibody (ANA) titers were all normal.

**Figure 1:**
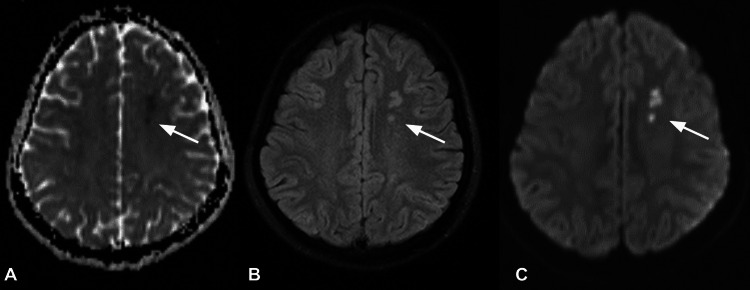
ADC (A), FLAIR (B) and DWI (C) axial sequence in MRI brain showing multifocal acute watershed infarction in left centrum semiovale region (white arrow) ADC: Apparent Diffusion Coefficient; DWI: Diffusion-Weighted Imaging; FLAIR: Fluid Attenuated Inversion Recovery; MRI: Magnetic Resonance Imaging

**Figure 2:**
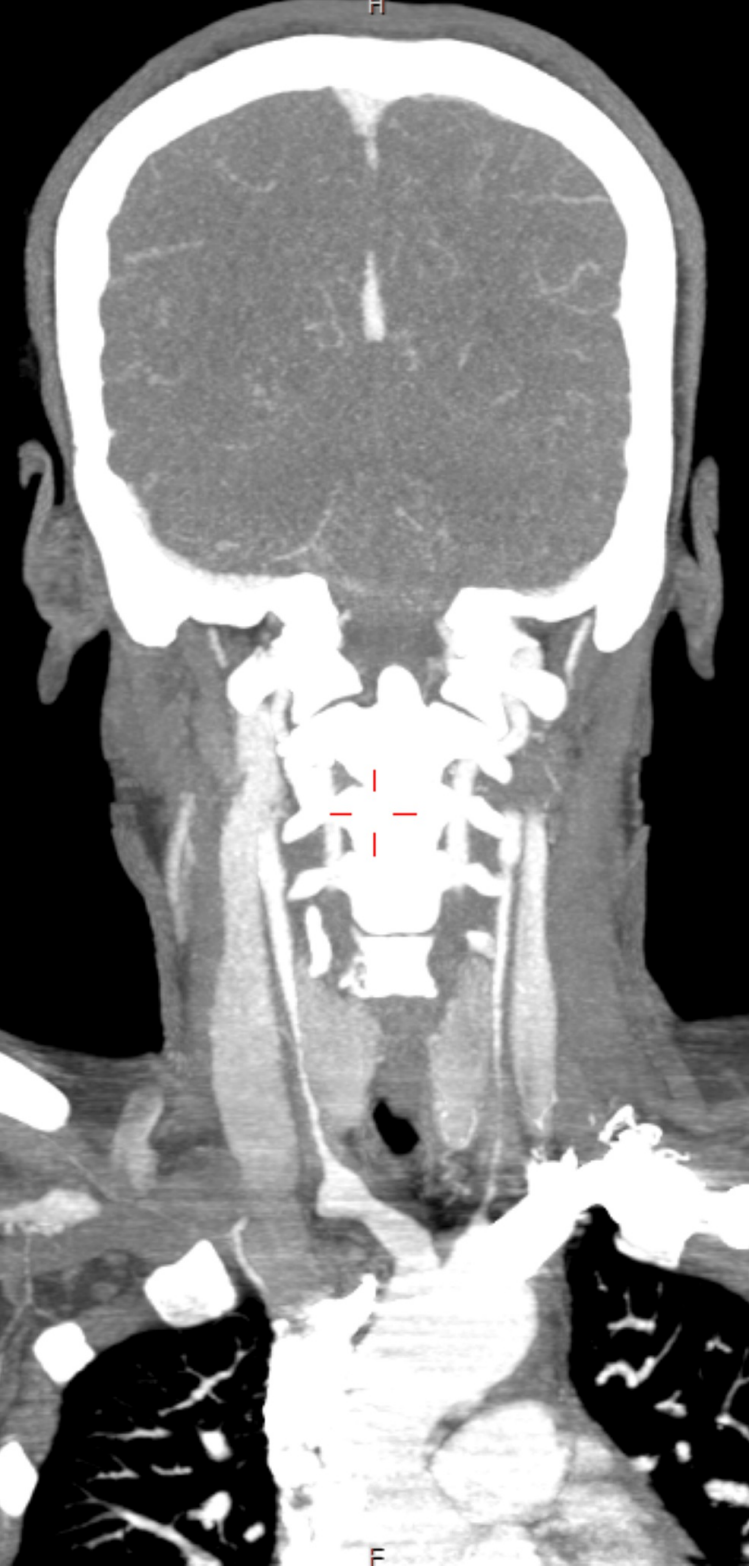
A computed tomography angiography of the head and neck showing severely attenuated bilateral common carotid arteries from their origin, as well as smooth wall thickening of the aortic arch and its branches

Brain magnetic resonance imaging (MRI) on ADC, FLAIR, and DWI revealed multifocal acute watershed infarction in the left centrum semiovale region (Figures 1A-1C) but no stenotic or occlusive lesions in the intracranial circle of Willis arteries and extracranial carotid and vertebral arteries. A computed tomography (CT) angiography of the head and neck, on the other hand, revealed severely attenuated bilateral common carotid arteries from their origin, as well as smooth wall thickening of the aortic arch and its branches, confirming TA (Figure 2). A 2D echocardiogram revealed concentric left ventricular hypertrophy, grade 1 diastolic dysfunction, and a normal ejection fraction. Electroencephalogram evidence pointed to epilepsy. A chest x-ray revealed no abnormalities. On CT, the abdomen and pelvis were unremarkable.

As a short-term treatment for the convulsions, 1,000 mg of the antiepileptic drug Levetiracetam was given intravenously (IV) and then orally and the patient was given five days of IV methylprednisolone 1,000 mg for an acute inflammatory reaction, followed by 50 mg oral prednisolone which was tapered over 15 days. Mycophenolate mofetil 500 mg twice a day was also used in her treatment which was continued for three months. Additionally, she received 75 mg of aspirin once daily, which was continued during follow-up. Her vision in the affected eye improved after two months of immunosuppressive treatment, and she had no recurrence of limb weakness or headache. A palpable radial pulse and elevated blood pressure were present in both arms. CRP and ESR levels were also found to be within normal ranges.

## Discussion

TA is a rare chronic vasculitis with an unknown cause that primarily affects the aorta and its primary branches [1]. Women are affected in 80% to 90% of cases, with onset ages ranging from 10 to 40 years. It is prevalent worldwide, with Asia having the highest prevalence [6,7].

The early inflammatory phase of TA is characterized by fatigue, malaise, fever, night sweats, joint aches, weight loss, and fainting. The secondary pulseless phase follows, which is marked by dyspnea, palpitation, headache, rash, hemoptysis, ulceration, and weight loss [3,8].

TA can also present with aneurysmal dilatation and resultant sluggish flow in common carotid and vertebral arteries, leading to ischemia and manifesting with neurological signs and symptoms [3,9]. ​Seizures can occur after early-or late-stage hemorrhagic stroke but are rare in acute ischemic stroke. Only a small percentage develop epilepsy in the future [10].

The common neurological symptoms of TA include dizziness and visual disturbance, followed by headache and ischemic stroke, epileptic seizures, and in rare cases, reversible posterior encephalopathy syndrome [11]. Though TA may be complicated with neurological manifestation, seizures, however, are rarely the first clinical manifestation [3,12]. Table 1 shows the criteria used by the American College of Rheumatology to classify TA. Other large vessel vasculitis, such as giant cell arteritis (GCA), systemic lupus erythematosus, syphilis, tuberculosis, and congenital connective tissue disorders, such as Marfan's syndrome, are in the differential diagnosis for TA [13].

**Table 1:** Criteria of American College of Rheumatology for the classification of Takayasu’s arteritis For purposes of classification, a patient shall be said to have Takayasu’s arteritis if at least three of these six criteria are present. The presence of any three or more criteria yields a sensitivity of 90.5% and a specificity of 97.8% [6].

Criteria	Definition
Age at disease onset (Years)	At the age of 40, the development of Takayasu's arteritis symptoms or findings.
Claudication of extremities	Development and worsening of fatigue and discomfort in muscles of one or more extremities while in use, particularly the upper extremities
Decreased brachial artery pulse	Pulsation of one or both brachial arteries is reduced.
Blood pressure difference >10 mmHg	A difference in systolic blood pressure of more than 10 mmHg between arms.
Bruit over subclavian arteries or aorta	Auscultation reveals a bruit over one or both subclavian arteries or the abdominal aorta.
Arteriogram abnormality	Arteriographic narrowing or occlusion of the entire aorta, its primary branches, or large arteries in the proximal upper or lower extremities that are not caused by arteriosclerosis, fibromuscular dysplasia, or other similar conditions: Typically, changes are focal or segmental.

Our patient was a young woman who was hospitalized with loss of consciousness followed by abnormal body movements due to cerebral hypoxia and vision loss due to retinal artery occlusion. We performed echocardiography with ophthalmoscopy, brain MRI, head and neck CT angiography, and carotid doppler imaging, and subsequently diagnosed with TA.

Antiepileptic treatment was found to be effective and may be considered a reasonable first step. The most effective drugs for TA are corticosteroids, but early detection and use of immunosuppressive agents are required to avoid vascular complications [7,14]. According to the 2021 ACR/VF Guideline, immunosuppressants such as prednisone and nonglucocorticoids such as methotrexate, tumor necrosis factor inhibitors, and azathioprine are used. Because it is well-tolerated, methotrexate is typically preferred for children [15]. In addition, mycophenolate mofetil is also safe and effective leading to a significant improvement in TA [16]. Aspirin is also recommended to lower the risk of ischemia events [15].

## Conclusions

This article aims to draw attention to rare diseases and atypical presentations, especially in the underdeveloped countries of Southeast Asia and seeks to raise awareness among physicians. TA is a large vessel vasculitis that can present with epileptic seizures. It is associated with significant morbidity and can be life-threatening in some cases. TA is linked to cardiovascular complications, stroke, and reno-vascular hypertension. Women with generalized weakness, vision loss, and systemic symptoms like headache with an absent radial pulse need to be evaluated for TA.
